# Induction of compulsive-like washing by blocking the feeling of knowing: an experimental test of the security-motivation hypothesis of Obsessive-Compulsive Disorder

**DOI:** 10.1186/1744-9081-1-11

**Published:** 2005-07-26

**Authors:** Erik Z Woody, Victoria Lewis, Lisa Snider, Hilary Grant, Markad Kamath, Henry Szechtman

**Affiliations:** 1 Dept of Psychology, University of Waterloo, Waterloo, Ontario, Canada; 2 Dept of Psychiatry and Behavioural Neurosciences, McMaster University, Hamilton, Ontario, Canada; 3 Dept of Medicine, McMaster University, Hamilton, Ontario, Canada

## Abstract

**Background:**

H. Szechtman and E. Woody (2004) hypothesized that obsessive-compulsive disorder results from a deficit in the feeling of knowing that normally terminates thoughts or actions elicited by security motivation. To test the plausibility of this proposed mechanism, an experiment was conducted to produce an analog of washing in obsessive-compulsive disorder by eliciting a scenario of potential harm and using hypnosis to block changes in internally generated feelings that would normally occur during washing.

**Results:**

Participants reacted with increased disgust, anxiety, and heart rate to their mental images of contamination and potential danger. As predicted, high but not low hypnotizable participants showed a significant prolongation of washing when change in feelings during washing was blocked hypnotically.

**Conclusion:**

Results show that blocking the affective signal that is normally generated during security-related behaviors, such as washing, leads to prolonged performance of these behaviors. This finding lends support to the plausibility of the proposed model of obsessive-compulsive disorder.

## Background

In obsessive-compulsive disorder (OCD), a sense of compulsion is associated with performing ritualistic thoughts or actions. There are two types of mechanism that might explain the intrusiveness and urgency characteristic of OCD symptoms. One possibility is that there is a pathological intensity of excitation in the system that initiates the particular thoughts or actions, such that they are elicited too readily and strongly [e.g., [1]]. A contrasting possibility is that there is a deficit in the system that normally terminates these thoughts or actions, such that they persist too long.

The idea that OCD symptoms stem from a pathologic intensity of excitation is intuitively appealing because it is consistent with the widespread notion of compulsion as a force that initiates behavior. However, Reed [[2], p. 127] found that only a tiny minority of OCD patients described their experience of compulsions in such a way. Instead, the great majority described their experience of compulsions in terms of an inability to stop – for example, "I keep wondering, and then I can't get it out of my mind," or "I can't move on because I can't convince myself that I've finished what I'm doing." Reed [[3], p. 384] concluded that "those who are trapped in a circle of repetitive behavior do not report that something forces them to *continue*, but that they lack something to make them *stop*."

Likewise, descriptive accounts of OCD behavior suggest that most patients engage in few but extended episodes of compulsive behavior during the day, rather than episodes of normal duration but excessive frequency [4]. Such a behavioral profile suggests a dysfunctional stop mechanism rather than activation mechanism.

### Conceptualizations of OCD as a Cognitive Disorder

Some conceptualizations of OCD have focused on the hypothesis that there is an underlying disorder of cognition. There are various ways a cognitive disorder might explain the inability to terminate thoughts and actions normally. For example, Reed [2] suggested that OCD symptoms may be the result of a central cognitive deficit in the defining of categories, in the determination of boundaries and limits, in the establishment of criteria, and in the allocation of class members. He argued that the obsessional style and engagement in rituals of these patients represent attempts to compensate for their cognitive inability to define and put closure on experiences. Similarly, Pitman [5] referred to this cognitive inability as a failure in the sense of task completion, and Pélissier and O'Connor [6] described it as a dysfunctional pattern of inductive reasoning.

Other recent explanatory models of OCD have also been strongly cognitive; for example, a major line of theorizing has implicated dysfunction in the metacognitive regulation of one's own stream of thoughts [7]. Accordingly, Salkovskis [8-10], Rachman [11,12], and Wells [13] have suggested a causative role for various dysfunctional beliefs that OCD patients appear to have about the meaning and implications of their conscious thoughts – for example, the belief that thinking something bad is virtually the same as actually doing it (thought-action fusion). In other words, OCD patients may have difficulty terminating thoughts and actions because they accord them exaggerated and perhaps irrational significance.

### OCD as a Disorder of Security Motivation

However, such cognitive models do not seem to account well for some of the key features of OCD. In particular, a striking feature of the disorder is the inability to feel reassured by seemingly obvious and compelling information from the senses. For example, although compulsive hand washers know objectively that their hands look clean, they cannot generate the normal subjective conviction that they are truly clean, and so continue to wash [14].

Somewhat in contrast to cognitive approaches, we have recently proposed a theory of OCD that focuses on its motivational underpinnings [15]. According to this theory, OCD patients are haunted by a sense of anxiety because their particular concerns and behaviors are invoked by a potent special motivation that handles potential threats to existence (e.g., predation) and protection from harm. Because the concerns of the system are potential rather than imminent threats, this motivational system is open-ended (in the sense that logical certainty about the absence of potential threat is unattainable); consequently, the system is not under immediate environmental control. Due to this lack of a terminating signal in the environment, goal completion in this system is normally signaled by an endogenously generated terminator (experienced as a feeling of knowing or task accomplishment), but OCD patients either cannot generate this emotional signal or it is inadequate to inhibit the invoked motivation.

To denote the particular feeling of knowing that serves as an essential terminator of the species-specific motivation concerned with protection from harm, we coined the term "yedasentience," [16] from the Hebrew yeda = knowing and Latin sentire = to feel. Our core hypothesis may then be stated as follows [[15], p. 116]:

An internally generated feeling of knowing (termed *yedasentience*) provides a phenomenological sign of goal-attainment and has as its consequence the termination of thoughts, ideas or actions motivated by concerns of harm to self or others. Failure to generate or experience this feeling produces symptoms characteristic of OCD.

The purpose of present study was to test the possibility that dysfunction of such a feeling of knowing is a plausible mechanism for OCD-like behavior. Our experimental approach was to block this feeling and see if the blockage leads to OCD-like behavior – specifically, prolonged washing. In this way, we hoped to demonstrate that we could temporarily create in non-patient individuals an OCD-like profile of behavior.

### Design of the Experiment

To produce an experimental analog of OCD washing, we needed to address two major issues. The first was how to create a sense of potential harm and thus elicit the security motivation underlying OCD behavior.

In our pilot studies, we initially tried to generate a sense of potential harm by using the methodology of Jones and Menzies [17]. In this approach, the experimenter asks participants to immerse their hands in a noxious mix of wet dirt and other materials and tells them, "For ethical reasons I should inform you that in this sort of procedure *there is always the possibility *of picking up bacteria that will result in serious illness" [[17], p. 123]. However, debriefing revealed that our participants did not find this danger protocol credible, perhaps at least in part because the experiment was taking place in a university hospital (and, of course, they also knew it had received ethical approval). Hence their experience lacked the appropriate emotional quality and significance.

Therefore, instead of providing a physical stimulus, we allowed the participants to use their imagination and recall their own experience of being in contact with something contaminated. We instructed them to imagine not only this specific experience but also the emotional reactions, such as disgust, that would accompany it. The use of such mental images as stimuli is consistent with research showing that imagination activates many of the same neural systems as are evoked by actual stimuli. Indeed, based on this research Kosslyn [18] has advanced the *reality simulation principle*:

"An object seen in a mental image can have the same impact on the mind and body that the actual object would have. ... Once the brain systems are engaged, they don't know where the impetus came from. This means that they can produce the same effects whether you activated it endogenously (from information in memory) or exogenously (from looking at something)."

The second major issue in designing the experiment was how to block yedasentience, the endogenous signal that we hypothesize normally terminates security-motivation-driven washing behavior. We used hypnosis for this purpose, because in people who are high in hypnotic responsiveness this technique permits the induction of far-reaching alterations in the sense of reality, independent of objective sensory input [e.g., [19-21]]. For example, individuals high in hypnotic suggestibility are able, under hypnosis, to experience hallucinations in a variety of sensory systems; in addition to such positive hallucinations, they are also capable of experiencing striking negative hallucinations – that is, not experiencing something actually present to their senses [e.g., [22]]. In addition, with hypnosis one can dissociate emotional experience from sensory qualities, as shown for example in the hypnotic manipulation of the emotional experience of pain independent of the perception of its sensory qualities [23].

Thus, using hypnosis in appropriately preselected participants, it is quite possible to dissociate subjective experience from the objective input available to the senses, and independently manipulate subjective convictions. It is worth stressing that we are using hypnosis as an empirical method to obtain a preparation suitable for testing the working hypothesis; we are not asking whether high hypnotic ability does or does not make one prone to OCD.

In summary, our experiment attempted to produce an analog of OCD washing by eliciting the feeling of potential harm and then blocking the changes in feeling that would normally occur during washing. It follows from the security-motivation hypothesis of OCD that the combination of these two conditions should yield prolonged washing. In addition, we included both high and low hypnotically responsive participants in the experiment. Because blocking changes in feeling should only be possible for highly hypnotizable participants, the low participants serve as a control for demand effects (e.g., participants merely behaving differently because it was directly implied that they should). Thus, the results of the experiment should yield a three-way interaction involving potential harm, blocking of change in feeling, and hypnotic susceptibility.

## Method

### Overview

Participants preselected as High or Low in Hypnotizability came to the lab to take part in a study described as addressing the physiological changes that accompany everyday behaviors and emotions. Heart-rate electrodes were attached to participants, they engaged in an initial hand washing to familiarize them with the sink set-up, and then they were hypnotized. Participants in the Potential Harm Suggested condition were instructed to imagine an emotional experience of touching a disgusting, contaminated object, whereas those in the Potential Harm Absent condition were asked to imagine an emotional experience of calm and relaxation. Next, participants in the Yedasentience Blocked condition were told that when they washed their hands they would not experience a sense of satisfaction, whereas those in the Yedasentience Not Blocked condition were told they would experience the usual sense of satisfaction. The main dependent variable was the duration of the subsequent hand-washing behavior.

### Participants

The sample consisted of 96 female and 53 male university students and other individuals who responded to notices posted in the teaching and hospital buildings of McMaster University or to recruitment in undergraduate classes. Participants were either paid or given partial course credit. All prospective participants were pre-screened with the Waterloo-Stanford Group C Scale (WSGC; [24,25]) or, in a minority of cases, the Harvard Group Scale of Hypnotic Susceptibility, Form A (HGSHS:A; [26]). For inclusion in the study, participants were required to score either high (9–12) or low (0–3) in hypnotizability on these scales. To maximize statistical power in the high hypnotizable cells, approximately one-third of participants selected were low hypnotizable (58, or 38%) and two thirds were high hypnotizable (91, or 63%). As a consequence, the four experimental conditions for low hypnotizables have a range of 14 to 15 participants each, and the four conditions for high hypnotizables have a range of 21–24. The mean age of the participants was 25, with a range from 16 to 67 years. Of the 149 participants, 83 (55.7%) were 16 to 19 years of age, 36 (24.2%) were 20 to 28, and 30 (21.1%) were over 30. The study received ethics approval at both McMaster University and the University of Waterloo.

It may be noted that hypnotic susceptibility has a modest inverse relation with non-dissociative psychopathology, such as mood and anxiety disorders [e.g., [27]]. Thus, it is unlikely that the high hypnotisability group would inadvertently consist of individuals with more OCD-like tendencies prior to the experimental manipulations. Likewise, the modest relationship does not preclude the generalization of obtained findings to OCD patients.

### Apparatus

Hand washing took place at a sink installed with an automatic faucet and an automatic soap dispenser, both activated by the proximity of hands. The faucet was preset to deliver a flow of water at a constant rate and temperature that did not vary across participants; the delivery of soap was similarly constant. A video camera (Panasonic AG-456UP) mounted directly over the sink, approximately one meter above it, recorded all washing episodes during the experiment onto a videotape (Panasonic (PV-VS4821-K). The camera lens was zoomed to capture a clear view of hands, illuminated by a 500 W type "T" halogen light bulb. A second video camera (Hitachi VM-7500LA) mounted at another location away from the sink captured a view of the entire room and provided a record of the whole experimental session. For recording of heart rate, the ECG signal was digitized at a sampling rate of 500 Hz using a 12-bit analog-to-digital converter (DATAQ, Akron, Ohio, U.S.A.) connected to an IBM-compatible PC; the ECG signal was displayed on the computer monitor throughout the session and stored on a hard disk at the defined periods; the mean heart rate during each recording period was later calculated using a QRS complex detection algorithm.

### Procedure

Participants took part in the study individually and remained seated in a comfortable swivel chair throughout its duration. To begin, the experimenter provided the following rationale:

"As we experience emotions, there are corresponding changes in our body. In this experiment, we want to study that connection between emotions and these bodily changes. Hence, one of the things I'm going to do is to attach you to this heart rate monitor that will sensitively measure changes in your body."

"Another important aspect of emotion is that people differ considerably from one another in their emotional responses. In this experiment we want to find out your particular pattern of response. Accordingly, I will ask you to engage in some everyday behaviors, such as washing your hands. I will also make some suggestions about your feelings. I will record your underlying responses for three minute periods between each of these behaviors or suggestions. In addition, we need to videotape all the participants so that we can review their overt behavior."

"Finally, as you know, I will be hypnotizing you at the beginning of the experiment. The hypnosis allows you to respond to the suggestions about your feelings. It also helps you to clear your mind and relax your body. Under these conditions, we can get a much better baseline against which to sensitively measure subtle emotional changes."

After the experimenter had attached the heart-rate electrodes to the skin over participants' collarbones and lower rib, she instructed them to turn to the sink and wash their hands, thus familiarizing them with the washing set-up and procedure, including a tap activated by an automatic sensor, an automatic liquid soap dispenser, and a supply of paper towels for drying. Once participants finished washing, the experimenter instructed them to move as little as possible for 3 minutes, with hands resting in lap and eyes closed, and during this period, their baseline heart rate was recorded.

Next, the experimenter administered each participant the standardized hypnotic induction from the WSGC, which includes instructions for focusing attention, eyes closing, relaxation, and count-based deepening. At the conclusion of the induction, the experimenter asked participants to remain deeply hypnotized, as still as possible with their eyes closed, and their heart rate was recorded for another three-minute period.

At this point, participants in the Potential-Harm-Suggested condition were given the following instructions:

"I want you to think of an emotional experience that I am about to describe. I want you to think of something you could touch that you would find really disgusting. Something that could be contaminated with germs and bacteria. Something like feces ... or dirty toilet water ... or vomit ... or worms ... bugs – whatever you find disgusting. When you think of that object, I want you to imagine that you have touched it – something that is disgusting and may be contaminated with germs and bacteria. You feel disgusted because you touched something that could be contaminated with germs and bacteria. Think how disgusted and contaminated you feel after touching this object."

"Now keep your eyes closed and your hands resting in your lap. Just keep them there, without further movement, and with your eyes closed, for three minutes while we take a heart rate recording. During the 3 minutes, I want you to think about how disgusted and contaminated your hands make you feel. Throughout this time, remain hypnotized, with your eyes closed, attending to how disgusted your hands make you feel."

In contrast, participants in the control (Potential-Harm-Absent) condition were instead given the following instructions:

"I want you to think of an emotional experience that I am about to describe. I want you to think of something that you could do that would be very relaxing. Something that would make you calm and relaxed. Something like reading a book ... or watching TV ... listening to quiet music – whatever you find relaxing. When you think of it, I want you to imagine that you are doing it – something that is relaxing and calming. You feel pleasantly relaxed and calm because this is something that you enjoy doing. Think of how relaxed and calm you feel."

"Now keep your eyes closed and your hands resting in your lap. Just keep them there, without further movement, and with your eyes closed, for three minutes while we take a heart rate recording. During the 3 minutes, I want you to think about how calm and relaxed you feel. Throughout this time, remain hypnotized, with your eyes closed, attending to how calm and relaxed you feel."

After heart rate had been recorded for another three-minute period, participants were very briefly reminded of the kind of experience they were supposed to keep in mind, either "how disgusted and contaminated your hands make you feel" or "how calm and relaxed you feel." Next, participants in the Yedasentience-Blocked condition were given the following instructions: 

"Now listen closely to my words, because this is very important. As you know: usually when you wash your hands there is a feeling of satisfaction that comes with it... However, now when you wash your hands, you will find that you do not experience that feeling of satisfaction. There will be a lack of satisfaction as you wash your hands."

"Okay, now open your eyes and turn to face the sink. Now go ahead and wash your hands with soap. Continue to think of how disgusted and contaminated your hands make you feel. Keep in mind that as you wash your hands, you will feel little or perhaps even no sense of satisfaction. The usual sense of satisfaction from washing your hands will be weak, or even absent."

In contrast, participants in the control (Yedasentience-Not-Blocked) condition were instead given the following instructions:

"Now listen closely to my words, because this is very important. As you know: usually when you wash your hands there is a feeling of satisfaction that comes with it... And when you wash your hands, you will find that you experience that feeling of satisfaction as you normally would. There will be a normal sense of satisfaction as you wash your hands."

"Okay, now open your eyes and turn to face the sink. Now go ahead and wash your hands, with soap. Continue to think of how disgusted and contaminated your hands make you feel. Keep in mind that as you wash your hands, you will feel a normal sense of satisfaction. You will experience the usual sense of satisfaction from washing your hands."

Participants then completed the washing and drying of their hands, which was recorded by video camera to allow accurate, objective determination of response duration. The experimenter next asked participants to close their eyes and make themselves comfortable in the chair, deeply hypnotized, with hands resting in lap, while heart rate was recorded for the last three-minute period.

In the last stage of the study, the experimenter carefully cancelled potentially disturbing suggestions (having touched something disgusting, and the inability to experience a sense of satisfaction from washing hands) for those participants who had been given them, and all participants were given another opportunity to wash their hands to show that they were now "clean and normal." Next, the experimenter brought participants out of hypnosis using the count-down procedure from the WSGC. After removing the electrodes she asked participants to describe the emotional experience they had been thinking of during the middle part of the experiment. Participants then filled out a brief questionnaire about their feelings during the study. Specifically, they rated their feelings when they were thinking of an emotional experience on five-point scales, from "not anxious" to "very anxious, and from "not disgusted" to "very disgusted." They also rated the extent to which they had experienced a sense of satisfaction while washing their hands in the middle part of the experiment, from "not at all" to "very satisfied." Finally, all participants were fully debriefed, thanked for their participation, and paid or given credit.

### Measurement of Dependent Variables

The duration of washing was measured from the videotapes as the amount of time in seconds from the beginning of hand washing, when participants made the initial contact with soap or water, to its end, when participants removed their hands from the flow of water just prior to drying them with paper towels. Due to technical reasons associated with recording a measurable ECG signal, somewhat fewer data are available for heart rate than for the duration of washing.

## Results

### Duration of Washing

The main dependent variable in this study is the duration of the hand washing following the experimental manipulations. An analysis for outliers indicated that three of the response durations fell more than 3.5 standard deviations above the overall mean, and therefore these data points were omitted from the following analysis. All three outliers occurred in the potential-harm, yedasentience-blocked cell (the one hypothesized to lead to exaggerated response duration); two of the participants were high hypnotizable and one was low hypnotizable. In two of the cases, the experimenter stopped the participant from engaging in further hand washing after about 5 minutes by saying, "That's fine." The other outlying response duration was also almost 5 minutes (253 s); in comparison, the next longest response duration in the sample was 72 seconds.

We performed a three-way between-subjects analysis of covariance of the duration of washing, using baseline washing time as the covariate. The factors were Hypnotizability (high vs. low), Potential Harm (present versus absent), and Yedasentience (blocked vs. not blocked). This analysis yielded the predicted three-way interaction, *F*(1, 137) = 7.125, *p *= .009. Other effects that were statistically significant were the two-way interactions of Hypnotizability by Yedasentience, *F*(1, 137) = 4.285, *p *= .04, and of Potential Harm by Yedasentience, *F*(1, 137) = 4.926, *p *= .028, and all the main effects: Hypnotizability, *F*(1, 137) = 13.908, *p *< .001; Potential Harm, *F*(1, 137) = 43.004, *p *< .001; and Yedasentience, *F*(1, 137) = 5.341, *p *= .022. Altogether, these effects, along with baseline washing, explained 52% of variance. An analysis of these factors together with Gender yielded no significant effects for Gender or its interaction with any other factors.

**Figure 1:**
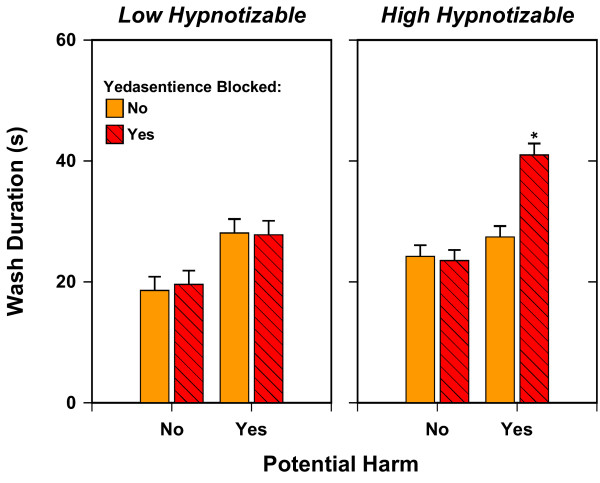
**Adjusted mean washing duration as a function of Hypnotizability, Potential Harm, and Blocking of Yedasentience**. Mean with an asterisk is significantly different from every other mean, p < .05. The combination of Potential Harm and blocked Yedasentience yielded prolonged hand washing in the highly hypnotizable participants, compared to all other conditions.

Figure 1 shows the adjusted means for this analysis. With regard to the significant three-way interaction, it is evident that blocking yedasentience significantly (*p *< .05) increased response duration only in the predicted cell, when potential harm had been suggested to high-hypnotizable participants. In contrast, blocking yedasentience had negligible and insignificant effects on response duration when potential harm was not suggested to highs, and when potential harm was suggested or not to lows. This pattern of results confirms the main hypothesis of the study. Also of some interest, the significant main effect of Potential Harm, together with the lack of any significant Hypnotizability by Potential Harm interaction, indicates that the suggestion of potential harm tended to increase washing time for all participants, regardless of their level of hypnotizability: For no suggestion of potential harm, the mean was 21.48 s, *SE *= 1.02, whereas for suggestions of potential harm, it was 31.07 s, *SE *= 1.04.

### Self-Reported Feelings

#### Disgust and anxiety

On five-point scales, participants rated the levels of disgust and anxiety they had felt after being asked to think of an emotional experience but before their subsequent hand washing. A three-way analysis of variance was performed on disgust, again with the factors Hypnotizability (high vs. low), Potential Harm (present versus absent), and Yedasentience (blocked vs. not blocked). This analysis yielded a significant Hypnotizability by Potential Harm interaction, *F*(1, 138) = 14.377, *p *< .001, and also significant main effects for both these factors: Hypnotizability, *F*(1, 138) = 10.460, *p *= .002; and Potential Harm, *F*(1, 138) = 262.784, *p *< .001. Together, the effects explained 71% of the variance in disgust ratings. The corresponding analysis of anxiety ratings, explaining 41% of the variance, yielded the same three significant effects: the Hypnotizability by Potential Harm interaction, *F*(1, 139) = 8.602, *p *= .004, and the main effects for Hypnotizability, *F*(1, 139) = 5.210, *p *= .024, and Potential Harm, *F*(1, 139) = 62.342, *p *< .001.

**Figure 2:**
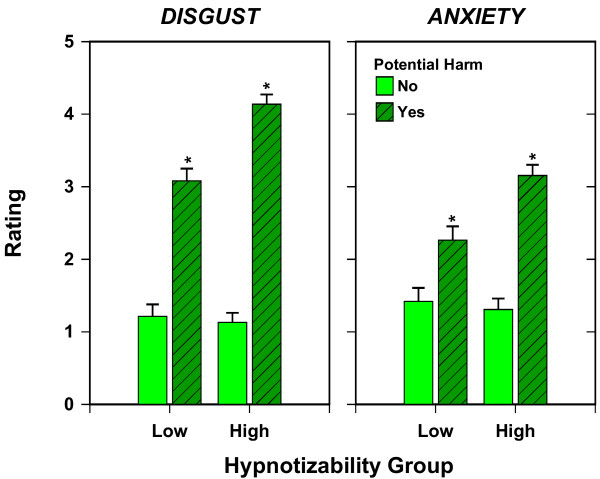
**Disgust and Anxiety as a function of Hypnotizability and Potential Hrm**. Means with an asterisk are each significantly different from the adjacent mean for No Potential Harm, p < .05. The suggestion of Potential Harm was effective in generating higher self-ratings of Disgust and Anxiety in both Low and High Hypnotizable participants, although significantly more so in the High Hypnotizable participants.

Figure 2 shows the means for Hypnotizability by Potential Harm for both disgust and anxiety. The manipulation of potential harm significantly (*p *< .05) increased disgust and anxiety levels for both low and high hypnotizable participants, indicating the success of this manipulation. However, the significant interactions indicate that the increases in disgust and anxiety were significantly greater for high hypnotizable participants than for their low hypnotizable counterparts.

#### Satisfaction while washing hands

**Figure 3:**
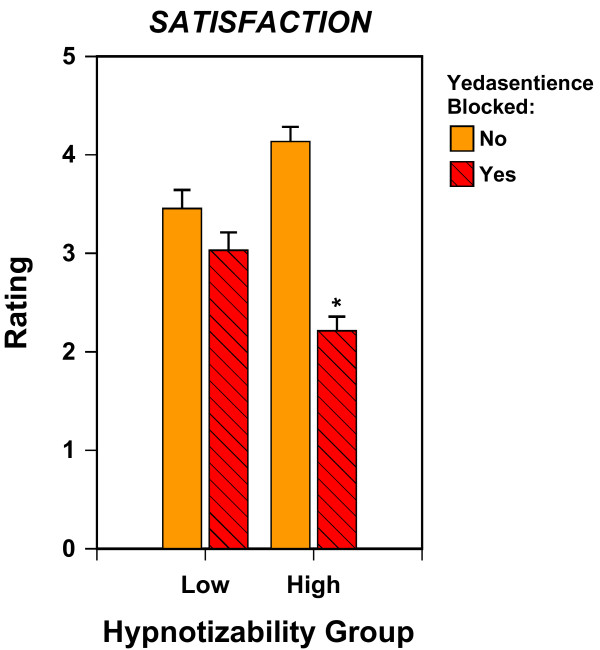
**Satisfaction as a function of Hypnotizability and Blocking of Yedasentience**. Mean with an asterisk is significantly different from the adjacent mean for No Blocking of Yedasentience, p < .05. Blocking Yedasentience significantly reduced self-ratings of satisfaction during the hand-washing in the High Hypnotizable participants, but not in the Low Hypnotizable participants.

Also on a five-point scale, participants rated the level of satisfaction they had experienced while subsequently washing their hands. The corresponding three-way analysis of variance of these ratings, explaining 40% of the variance, yielded a significant Hypnotizability by Yedasentience interaction, *F*(1, 139) = 20.246, *p *< .001, and a significant main effect of Yedasentience, *F*(1, 139) = 49.781, *p *< .001. Figure 3 provides the associated means. For the high hypnotizable participants, blocking yedasentience significantly (*p *< .05) reduced their experience of satisfaction while washing their hands; whereas for the low hypnotizable participants, this effect was negligible and statistically insignificant. The implication is that, as anticipated, only high hypnotizables can effectively enact the suggestion to block yedasentience.

#### Heart Rate

**Figure 4:**
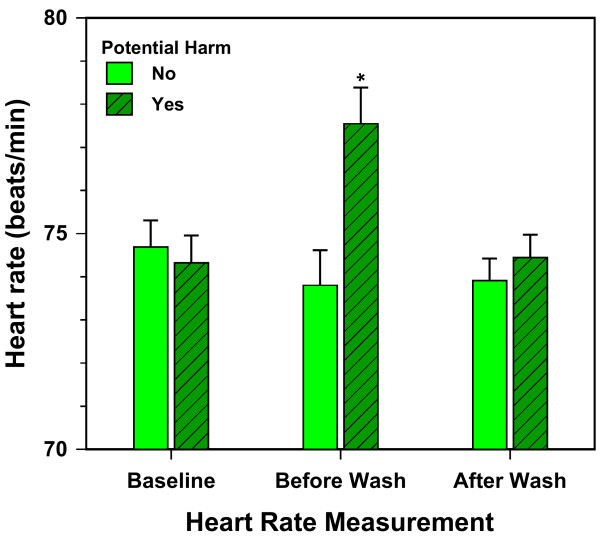
**Heart Rate as a function of Time of Measurement and Potential Harm**. Mean with an asterisk is significantly different from every other mean, p < .05. The suggestion of Potential Harm increased participants' heart rates compared to the control suggestion; this increase disappeared once the participants had washed their hands.

The study also included a more covert index of how participants were feeling, namely their heart rate. We submitted the heart-rate data to a four-way mixed-model analysis of covariance, using baseline heart rate as the covariate. The three between-subject factors were Hypnotizability (high vs. low), Potential Harm (present versus absent), and Yedasentience (blocked vs. not blocked). The within-subject factor was Trials, with three times of measurement: Trial 1 was measured just after the hypnotic induction; Trial 2 was measured just after the suggestion of an emotional experience (e.g., a situation of potential harm); and Trial 3 was measured just after the completion of hand washing. This analysis yielded one significant effect, the two-way interaction of Trials by Potential Harm, multivariate *F*(2, 117) = 5.803, *p *= .004, which explained 9% of the variance (Wilk's Lambda = .910). Figure 4 shows the relevant means. The mean for Trial 2 in the potential-harm-suggested condition is significantly higher (*p *< .05) than each of the three other means, which in turn do not differ significantly from one another. Thus, the suggestion of an experience of potential harm increased participant's heart rates, whereas the control suggestion of a positive experience did not; in addition, this potential-harm-related increase dissipated fully once the participants had been allowed to wash their hands. (Note that it makes sense for the experimental factor of Yedasentience not to be involved in this effect: Its manipulation took place between Trial 2 and Trial 3, and heart rate at Trial 3 was measured after the completion of handwashing, when participants had been able to take as long as they wanted to clean their hands.)

## Discussion

Although high hypnotizables showed a particularly strong emotional response to their mental images of contamination and potential harm, all participants tended to respond with increased disgust and anxiety. In addition, all participants, regardless of their level of hypnotizability, tended to react to their images of potential harm with elevated heart rate and increased washing time, and this elevated heart rate returned to baseline when they had washed. Taken together, these self-report, heart-rate, and behavioral data indicate that both high and low hypnotizable participants succeeded in imagining a situation of potential harm in a vivid and involving way. This is an essential precondition for the meaningfulness of the central manipulation of the experiment, which was the blocking of yedasentience.

The effect of the yedasentience-blocking suggestion was highly specific: It had the predicted effect of prolonging the duration of washing only in the predicted condition, in which potential harm had been suggested to high-hypnotizable individuals. This key result supports our hypothesis that the dysfunction of such a feeling of knowing is a plausible mechanism for OCD-like behavior.

The pattern of results obtained also helps to discount certain alternative explanations of the results. For example, although we did not directly tell participants to wash longer, it might be argued that we simply implied it in the suggestion for a lack of a feeling of satisfaction. However, only the high hypnotizables showed prolonged washing in response to this suggestion, and they showed it only after potential danger had been invoked. Thus, their extra washing would appear to be an integrated, natural response to the blocking of yedasentience, rather than merely some reflection of demand characteristics. Similarly, another possible alternative explanation would be that the yedasentience-blocking suggestion acted inadvertently as an additional suggestion about the state of dirtiness of the participants' hands. However, contrary to such an interpretation, the effects of the Potential Harm and Yedasentience manipulations were not additive: For high hypnotizables, when yedasentience was not blocked, potential harm had no significant effect on washing time, and when potential harm was low, the blocking of yedasentience had no effect on washing time.

One might also question whether this increase in washing time, which was fairly modest in magnitude (about 20 s), was sufficiently long to represent an analogue of OCD-like washing. The 20-s increase needs to be put into perspective: It may be compared with the 42-s increase due to a high-danger manipulation that Jones and Menzies [17] obtained in the top 10% of scorers on an OCD-screening instrument, who had put their hands for 5 minutes in a garbage can of dirt, animal hair, raw meat, and household food scraps. In addition, it is noteworthy that in our study the participants' hands were never actually dirty (indeed, they had just been washed a few minutes previously). Finally, it is worth mentioning that three participants showed a far more prolonged response to the yedasentience-blocking suggestion, continuing to wash their hands for about 5 minutes, or possibly longer if they had not been stopped. What made these participants different from the others in this study is unknown, but it is relevant that they appeared quite anxious and uncomfortable during their hand washing.

### Limitations of the present study

There are two important limitations of the present study. First, the study pertains most directly to an understanding of compulsive behavior rather than obsessive thoughts. Second, the study addresses only one form of compulsive behavior, namely, washing, but there are other kinds of compulsive behaviors such as checking or hoarding. Nevertheless, it is important to note that the underlying model addresses a broad range of OCD phenomena, including obsessional symptoms, as discussed elsewhere [15,28].

Other potential limitations of the present study merit attention. Because hypnosis is sometimes considered to be an altered state of consciousness, it could be argued that washing behavior in this state has limited relevance to the behavior of OCD patients. For example, it might be thought that hypnosis would interfere with the experience of anxiety that characterizes the experience of OCD patients. However, as the presented self-reports (Figure 2) and heart rate data (Figure 4) clearly showed, the participants in the relevant groups did report anxiety in response to the suggestion of potential harm and this anxiety dissipated when they washed their hands. In fact, the state of hypnosis did not limit the extent of anxiety as evidenced by the observation that in the high hypnotizable participants anxiety levels were just as high as in low hypnotizables. Thus, the state of hypnosis is not incompatible with the experience of anxiety. Similarly, it might be thought that participants in hypnosis become incapable of making conscious decisions. However, as many studies have indicated, such a view is incorrect [29]. Overall, the demonstration of the effects of yedasentience blockade under hypnosis should apply to similar behavioral effects of yedasentience blockade in OCD patients.

Another potential limitation is that the study lacks a manipulation check for the success of yedasentience blockage. Two pieces of data address this issue. First, Figure 3 shows that self-ratings of satisfaction are consistent with the intended purpose of the manipulation to block yedasentience. Second, Figure 1 illustrates that the experimental manipulation produced the expected 3-way interaction, again providing support for the effectiveness of the manipulation. Thus, the effectiveness of yedasentience manipulation is not simply assumed and in fact the findings noted above constitute the empirical evidence that the manipulation was effective.

Finally, it might be objected that hypnotically induced behaviors are simply socially sanctioned role playing. The widely accepted control for this potential problem is to include low hypnotizable subjects, who are exposed to exactly the same role demands. The fact that the low hypnotizable participants in our study did not show the same response suggests that role playing is not the key explanation for the observed results.

### Implications for Future Research

Our instructions for imagining a scenario of potential danger were double-barrelled: They involved both the idea of potential danger (contamination) and the emotion of disgust. One may ask about the respective roles of these two aspects, and whether both are actually important in eliciting the relevant security motivation.

Along these lines, some recent work indicates that the emotion of disgust may be of special importance in OCD [30,30-33]. Nonetheless, although the relevance of disgust to compulsive washing seems clear, it is much more difficult to see its relevance to some other OCD behaviors – for example, compulsive checking.

There is some evidence that subtypes of OCD exist [34-36] and that checkers may be different from washers [37]. Accordingly, we would propose that the special role of disgust is as follows: If associated with the signal of potential danger there is an induced feeling of disgust, then washing responses are potentiated. Thus, although we would argue that the invocation of disgust is not the pathogenic characteristic of OCD (in our model, absence of yedasentience is pathogenic), the presence of disgust may be a factor that biases OCD symptoms towards washing compulsions. Substantiating the possibility that different subtypes of OCD may have different special emotions is an important topic for further research.

Similarly, another important task for future research is to show that blocking the feeling of knowing, as was done in the present experiment to elicit OCD-like prolongation of hand-washing behavior, can also elicit other major types of OCD-like behavior, including checking behavior. Such research could not only help to evaluate the generality of our findings, but also help to elucidate the differences between separable classes of OCD behavior – for example, whether there is another particular affect, paralleling the role of disgust in washing, that is specific for the invocation of checking behavior.

Finally, the security-motivation hypothesis of OCD has other important implications. For example, we have provided a detailed provisional model of its hypothesized neural underpinnings and speculated on its implications for treatment [15]. We hope the present demonstration of its plausibility stimulates wider interest in this hypothesis.

## Competing interests

The author(s) declare that they have no competing interests.

## Authors' contributions

EZW and HS were the principal investigators, who designed the experiment, performed the data analysis, and wrote the manuscript. VL, LS, and HG participated in the design of the study and helped to collect the data. MK assisted with carrying out the heart-rate investigations.
